# Comparison of posterior vitreous detachment-related and glaucomatous optic disc hemorrhage

**DOI:** 10.1038/s41598-023-32327-8

**Published:** 2023-03-27

**Authors:** Cho Jin, Gi Seok Park, Kyung Nam Kim, Mi Yeon Song, Young Hoon Hwang

**Affiliations:** 1 Department of Ophthalmology, Chungnam National University Hospital, 282 Munhwa-ro, Jung-gu, Daejeon, 35015 Republic of Korea; 2 Department of Ophthalmology, Samsung Medical Center, Sungkyunkwan University School of Medicine, Seoul, Republic of Korea

**Keywords:** Diseases, Medical research, Pathogenesis, Signs and symptoms

## Abstract

This study compared the characteristics of posterior vitreous detachment (PVD)-related and glaucomatous optic disc hemorrhage (DH). Fundus photographs of eyes with PVD-related DH (PVD group) and glaucomatous DH (glaucoma group) were reviewed. The shape, type, layer, location (clock-hour sector), and DH/disc area (DH/DA) ratio of DH were investigated. In the PVD group, DH presented as a flame (60.9%), splinter (34.8%), and dot or blot (4.3%) shape. However, most of the glaucomatous DH revealed a splinter shape (92.3%), followed by a flame shape (7.7%, *p* < 0.001). In the PVD group, the most common type of DH was cup margin type (52.2%), whereas, in the glaucoma group it was disc rim type (53.8%, *p* = 0.003). Both PVD-related and glaucomatous DH were most commonly observed in the 7 o’clock sector. In the PVD group, DH was also found in the 2 and 5 o’clock sectors (*p* = 0.010). Mean DH/DA ratio in PVD group (0.15 ± 0.19) was greater than in glaucoma group (0.04 ± 0.04, *p* < 0.001). PVD-related DH showed a higher frequency of flame shape, cup margin type, nasal location, and greater area compared to the glaucomatous DH.

## Introduction

Optic disc hemorrhage (DH) is defined as a hemorrhage appearing around the optic nerve head (ONH). As many studies have investigated the association between DH and glaucomatous damage, DH is widely considered a marker of the development and progression of glaucoma^1,2^. Therefore, the observation of DH in glaucoma may play an important role in the clinical decision for treatment initiation or escalation. However, DH also may occur in healthy eyes and conditions such as diabetes mellitus, optic disc drusen, ischemic optic neuropathies, vascular disease of the retina, systemic hypertension, leukemia, and systemic lupus erythematosus^3,4^. Hence, determining whether or not the DH is glaucomatous is essential.

Posterior vitreous detachment (PVD) is a separation of the vitreous from the retinal surface. The development of PVD can lead to a variety of complications, such as retinal tears, retinal detachment, vitreous hemorrhage, and retinal hemorrhage^5,6^. According to previous studies, DH is also found in conditions with PVD^7–13^. Given that both older age and higher myopia are common risk factors for the development of PVD and glaucoma^5,6,14,15^, the observation of PVD-related DH may require a differential diagnosis from glaucomatous DH.

To date, only a few studies reported clinical characteristics of DH accompanied by PVD without a comparison of glaucomatous DH^7–13^. This study aimed to compare the characteristics of PVD-related and glaucomatous DH to provide information necessary to differentiate glaucomatous DH from PVD-related DH and understand the mechanisms of DH.

## Results

### Clinical characteristics

A total of 23 (23 participants) and 52 (52 participants) eyes were enrolled in the PVD and glaucoma groups, respectively. The mean age and refractive errors were 57.5 ± 6.9 years and −2.31 ± 3.42 diopters for the PVD group and 59.9 ± 10.1 years and −1.29 ± 2.23 diopters for the glaucoma group. There was no significant difference in age, sex, history of diabetes, hypertension, refractive error, and IOP between the two groups (all *p* values > 0.05, Table 1). Eyes in the glaucoma group showed worse visual field indices than the PVD group (*p* < 0.001, Table 1).

**Table 1:** Clinical characteristics of eyes with posterior vitreous detachment-related optic disc hemorrhage (PVD group) and glaucomatous optic disc hemorrhage (glaucoma group).

	PVD group (n = 23)	Glaucoma group (n = 52)	*p* value
Age (years)	57.5 ± 6.9	59.9 ± 10.1	0.310*
Female (n)	11 (47.8%)	25 (48.1%)	0.591^†^
Diabetes (n)	0 (0%)	3 (5.8%)	0.327^†^
Hypertension (n)	2 (8.7%)	4 (7.7%)	0.602^†^
Refractive error (diopters)	−2.31 ± 3.42	−1.29 ± 2.23	0.128*
Intraocular pressure (mmHg)	13.8 ± 2.8	13.9 ± 2.9	0.983*
Visual field indices			
Mean deviation (dB)	−0.57 ± 0.94	−6.30 ± 6.06	< 0.001*
Pattern standard deviation (dB)	1.27 ± 0.50	7.20 ± 4.50	< 0.001*
Visual field index (%)	99.0 ± 0.9	82.5 ± 18.5	< 0.001*

*PVD* posterior vitreous detachment.

*Independent t-test.

^†^Fisher’s exact test.

### Features of disc hemorrhage

When features of PVD-related and glaucomatous DH were compared, PVD-related DH showed flame (60.9%), splinter (34.8%), and dot or blot (4.3%) shapes, while, most of the glaucomatous DH revealed splinter shape (92.3%), followed by flame shape (7.7%) (*p* < 0.001; Table 2). Distribution of flame or splinter shape of DH was significantly different in the post hoc analysis (*p* < 0.05). In the PVD group, the most prevalent type of DH was cup margin type (52.2%), while in the glaucoma group, disc rim type was most prevalent (53.8%), followed by peripapillary type (26.9%) and cup margin type (17.3%) (*p* = 0.003; Table 2). Distribution of cup margin or disc rim type of DH was significantly different in the post hoc analysis (*p* < 0.05). In the PVD group, DH was found in the subretinal layer in one eye, whereas subretinal DH was not observed in the glaucoma group (*p* = 0.307). Regarding the clock-hour sector location of DH, both PVD-related and glaucomatous DH were most commonly observed in the 7 o’clock sector, followed by the 6 (PVD group) or 11 (glaucoma group) o’clock sectors. In the PVD group, DH was observed in the 2 and 5 o’clock sectors, but not in the glaucoma group (*p* = 0.010). Distribution of DH in 2, 5, and 6 o’clock sectors was significantly different in the post hoc analysis (*p* < 0.05). The mean DH/DA ratio in the PVD group (0.15 ± 0.19) was significantly greater than that in the glaucoma group (0.04 ± 0.04) (*p* < 0.001; Table 2). Two eyes in the PVD group and three eyes in the glaucoma group had multiple hemorrhages. The distribution of eyes with multiple hemorrhages between the two groups did not show significant difference (*p* = 0.639; Table 2).

**Table 2:** Features of optic disc hemorrhage in eyes with posterior vitreous detachment-related optic disc hemorrhage (PVD group) and glaucomatous optic disc hemorrhage (glaucoma group).

	PVD group (n = 23)	Glaucoma group (n = 52)	*p* value
Shape (n)			< 0.001*
Splinter	8 (34.8%)	48 (92.3%)	
Flame	14 (60.9%)	4 (7.7%)	
Dot or blot	1 (4.3%)	0 (0%)	
Type (n)			0.003*
Lamina cribrosa type	0 (0%)	1 (1.9%)	
Cup margin type	12 (52.2%)	9 (17.3%)	
Disc rim type	3 (13.0%)	28 (53.8%)	
Peripapillary type	8 (34.8%)	14 (26.9%)	
Layer (n)			
Preretinal or retinal	23	52	0.307^‡^
Subretinal	1^§^	0	
Location (n, clock-hour)			0.010*
12	1 (4.3%)	2 (3.8%)	
1	0 (0%)	0 (0%)	
2	2 (8.7%)	0 (0%)	
3	0 (0%)	0 (0%)	
4	0 (0%)	0 (0%)	
5	2 (8.7%)	0 (09%)	
6	5 (21.7%)	3 (5.8%)	
7	7 (30.4%)	27 (51.9%)	
8	0 (0%)	4 (7.7%)	
9	2 (8.7%)	1 (1.9%)	
10	2 (8.7%)	3 (5.8%)	
11	2 (8.7%)	12 (23.1%)	
Disc hemorrhage/disc area ratio	0.15 ± 0.19	0.04 ± 0.04	< 0.001^†^
Multiple hemorrhages (n)	2 (8.7%)	3 (5.8%)	0.639*

*PVD* posterior vitreous detachment.

*Chi-square test.

^†^Independent t-test.

^‡^Fisher’s exact test.

^§^Disc hemorrhage was found both in retinal and subretinal layers within an eye.

## Discussion

DH is found both in PVD and glaucoma. In addition, PVD and glaucoma share common risk factors such as aging and myopia. As a result, DH in these conditions is challenging to differentiate. Our investigation demonstrated that PVD-related DH showed a higher prevalence of splinter shape, cup margin type, nasal location, and greater area compared to glaucomatous DH. These findings may provide information necessary to differentiate glaucomatous DH from PVD-related DH. To our knowledge, this is the first study to compare the characteristics of PVD-related and glaucomatous DH.

Based on the shape of DH, PVD-related DH showed flame (60.9%), splinter (34.8%), and dot or blot (4.3%) shapes. In contrast, glaucomatous DH was majorly splinter-shaped (92.3%). This difference may reflect the different mechanisms of the DH between PVD and glaucoma. PVD-related DH mainly occurs by mechanical injury to the superficial retinal vessels around ONH induced by traction force at the vitreopapillary attachment site^7–13^. Therefore, PVD-related DH may show various shapes according to the location and degree of traction. Among the three types, flame-shaped DH was most commonly observed in the PVD group. This may be explained by the development and spread of hemorrhage along with the vessel trajectory through separated space between the ONH surface and detached vitreous cortex. When DH is presented in the preretinal layer, it may be entrapped in the vitreous and show a dot or blot shape. In eyes with glaucoma, the rupture of small blood vessels at the level of the lamina cribrosa, ONH, or RNFL plays a major role in the development of DH^1,2^. Because of the anatomic alignment of the RNFL bundles, DH appears as a splinter-like area of bleeding which is radial and parallel to ONH and RNFL bundles, respectively. Therefore, DH in glaucoma mainly shows a splinter shape.

In the PVD group, the most common type of DH was the cup margin type, followed by the peripapillary type. In contrast, in the glaucoma group, disc rim type was most commonly found, followed by peripapillary, cup margin, and lamina cribrosa types. When PVD occurs, vitreopapillary traction is mainly made at the ONH margin where the vitreous is firmly attached^11^. Therefore, traction-induced vessel rupture may be more frequently found at the ONH margin or cup margin compared to the disc rim or lamina cribrosa. In glaucoma, lamina cribrosa is a key structure of axonal loss; therefore, DH can be found around it^16,17^. However, when PVD develops, traction force may not have a great effect on lamina cribrosa. As a result, no eye in the PVD group showed lamina cribrosa type DH.

The mean DH/DA ratio in the PVD group was approximately four times greater than in the glaucoma group. This finding suggests that DH size may be a useful discrimination point. Considering that PVD-related DH occurs between the ONH surface and liquefied or detached vitreous, it may have a lesser area restriction than glaucomatous DH. In addition, the greater traction force and involvement of larger vessels of ONH surface in PVD compared to the smaller vessels in lamina cribrosa or RNFL may attribute to this finding.

Regarding the clock-hour sector location of DH, both PVD-related and glaucomatous DH were most commonly found in the 7 o’clock sector, followed by the 6 (PVD group) or 11 (glaucoma group) o’clock sectors. In the PVD group, DH was also found in the 2 and 5 o’clock sectors, whereas in the glaucoma group, no DH was observed in these sectors. The glaucomatous change mainly occurs in inferotemporal and superotemporal areas of ONH^1,2^. Thus, it is predictable that DH in glaucoma is most found at the 7 and 11 o’clock sectors. In contrast, DH in PVD can occur at any location based on the location of traction at the ONH surface. According to previous studies, DH in PVD was also found in various locations including nasal areas of ONH^7–13^. Therefore, the presence of DH in the nasal area of ONH may indicate PVD-related DH rather than glaucomatous DH. Despite this difference, PVD-related DH was most commonly found in inferior or superior areas of ONH which is similar to glaucoma. Thus, the location may provide limited information.

Among the eyes with PVD-related DH, subretinal hemorrhage was found in the nasal area of one eye, whereas no eye showed subretinal hemorrhage in the glaucoma group. Previous studies also reported DH in a subretinal layer in conditions with PVD, especially in the nasal area of ONH^8,11,13^. When PVD-related DH develops, the traction may affect not only the surface of ONH but also the subretinal layer. Transmission of the shearing force through the layers of the retina may cause elevation of ONH and subretinal bleeding^11^. In contrast, glaucomatous changes mainly occur in lamina cribrosa, ONH, or retinal nerve fiber layer (RNFL) and do not directly affect the subretinal layer. Therefore, the presence of DH in a subretinal layer may imply PVD-related change rather than glaucomatous change. However, a single case in the present study may not be sufficient to explain the mechanism of subretinal hemorrhage.

This study has several limitations. The number of eyes in the PVD group was small, and only those with symptomatic PVD were enrolled. Enrollment of only eyes with PVD-related symptoms may bias the results toward more flame shaped hemorrhages if these are correlated with greater vitreopapillary traction. In addition, the diagnosis of PVD was mainly made by the PVD-related symptoms. In a previous study, a diagnosis of PVD was made by observing a Weiss ring, tractional retinal tear, and/or an optically empty posterior vitreous cavity in fundus examination^13^. However, the diagnosis of PVD on fundus examination may be limited if the PVD is minimal or partial, or if a Weiss ring is not visible. To acquire more detailed information on PVD and DH, such as the degree and location of traction to ONH, and the area and layer of DH, further studies using optical coherence tomography (OCT), OCT angiography, fluorescein angiography, or ultrasound imaging are needed. The number of eyes with an OCT device was insufficient for the analysis of OCT parameters. Therefore, a detailed investigation of the ONH structures, RNFL, and retinal ganglion cell layers was difficult.

Differentiating PVD-related and glaucomatous DH may prevent unnecessary glaucoma treatment. If the characteristics of DH are close to those of PVD-related DH and risk factors for glaucoma, such as high IOP or large cupping, are absent, observation without treatment may be possible. Despite the significant differences in DH characteristics between the PVD and glaucoma groups, there was still an overlap of characteristics. Additionally, PVD-related DH can develop in glaucomatous eyes. Therefore, it would still be difficult to completely differentiate between PVD-related and glaucomatous DH in a clinical setting.

In conclusion, PVD-related DH and glaucomatous DH observed in fundus photographs showed different features. As compared to glaucomatous DH, PVD-related DH more frequently showed flame shape, cup margin type, nasal location, and greater area. These findings may be helpful for the differential diagnosis of glaucomatous and PVD-related DH.

## Materials and methods

### Participants

This retrospective observational study was approved by the Institutional Review Board of the Chungnam National University Hospital, Daejeon, South Korea. All procedures were conducted in accordance with the Declaration of Helsinki. Because of the retrospective study design, the requirement for informed consent was waived by the Institutional Review Board of the Chungnam National University Hospital. In this study, participants with DH in association with PVD (PVD group) and glaucoma (glaucoma group) were included. In the PVD group, all patients presented to the clinic due to the sudden onset of PVD-related symptoms, such as floater, cobwebs, and flashing lights and presence of PVD-related DH from March 2021 to December 2021. PVD-related DH was defined as (1) the presence of DH on or around the ONH, (2) observation of DH within 1 month from the onset of PVD-related symptoms, and (3) absence of other possible causes of DH, including optic neuropathy or retinopathy. As a control, age, sex, and refractive error-matched glaucomatous eyes with DH were recruited from a previous study investigating the RNFL thickness changes using OCT following the observation of DH (glaucoma group)^18^. Glaucomatous DH was defined as the presence of DH in eyes with glaucoma without any other possible causes of DH.

### Ophthalmic examinations

All eyes underwent detailed ophthalmic examinations, including the measurement of refractive error, visual acuity, and intraocular pressure (IOP) using a Goldmann applanation tonometer, slit-lamp biomicroscopic examination, fundus photography, and automated visual field test using a Humphrey Field Analyzer (Carl Zeiss Meditec, Dublin, CA) with the 24-2 Swedish interactive thresholding algorithm. Visual fields with fixation losses and false-positive and -negative response rates < 20% were considered reliable.

The inclusion criteria were as follows: (1) age 18 years or older; (2) a best-corrected visual acuity of 20/40 or better; (3) a normal anterior segment on slit-lamp examination; (4) ONH without glaucomatous changes (PVD group); (5) ONH with glaucomatous changes, such as an increased cup-to-disc ratio and neuroretinal rim narrowing (glaucoma group).

The exclusion criteria were: (1) eyes with any retinal disease including diabetic retinopathy, hypertensive retinopathy, vascular diseases, such as a retinal vein or retinal artery occlusion, age-related macular degeneration, optic neuropathy except for glaucoma, or neurological disease; (2) patients with systemic disorders with the exception of diabetes or hypertension.

### Features of optic disc hemorrhage

**Figure 1:**
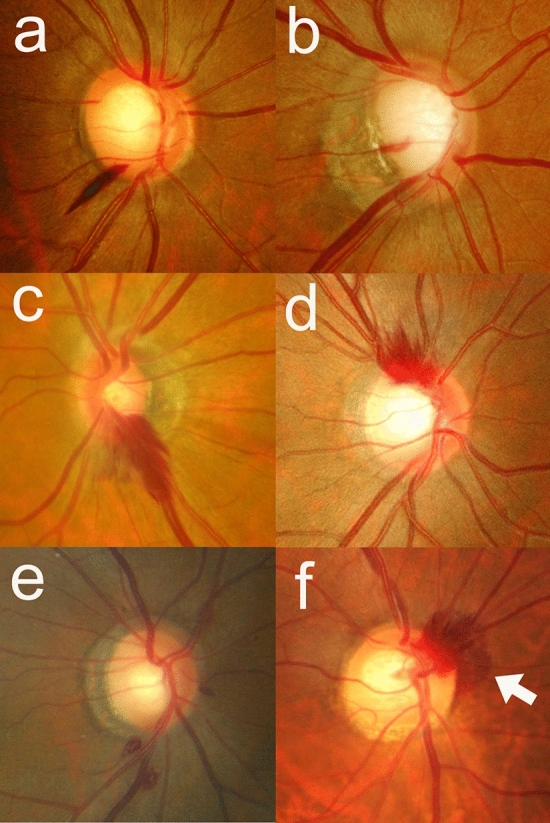
Representative fundus photographs of posterior vitreous detachment (PVD)-related and glaucomatous disc hemorrhage (DH). Splinter-shape glaucomatous DH with disc rim type (**a**), splinter-shape glaucomatous DH with lamina cribrosa type (**b**), flame-shape PVD-related DH with cup margin type (**c,d**), dot or blot-shape PVD-related DH with a peripapillary type (**e**), and flame-shape PVD-related DH with cup margin type (**f**) in combination with subretinal hemorrhage (white arrow) are presented.

**Figure 2:**
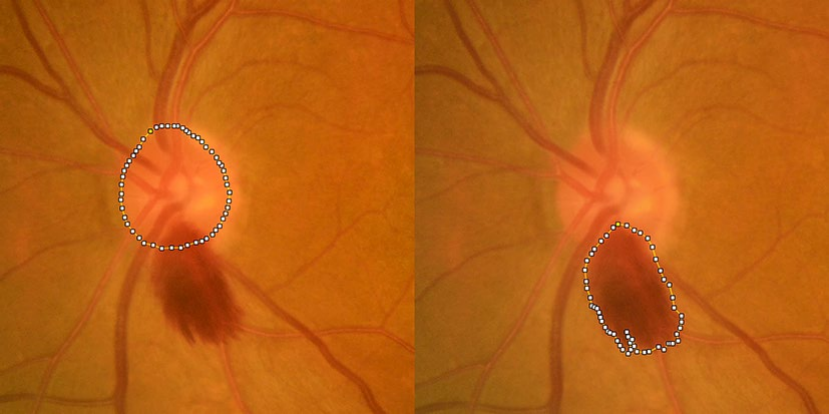
Measurement of disc area (DA) and disc hemorrhage (DH) area. Margins of the optic disc and disc hemorrhage are plotted and areas are measured. In this eye, the DH/DA ratio is 0.83.

By inspecting the fundus photographs, the following features of DH were investigated (Figs. 1 and 2): (1) shape (flame-shape, splinter-shape, dot or blot-shape); (2) type (lamina cribrosa type, cup margin type, disc rim type, peripapillary type according to the proximal location of DH^16,17^); (3) location (clock-hour sector, with the 12 o’clock sector at superior and 9 o’clock sector at the temporal margin of ONH); (4) layer (pre-retinal or retinal layer vs. subretinal layer); (5) DH/disc area (DH/DA) ratio. DH/DA ratio was defined as the ratio of DH and ONH areas measured using ImageJ (National Institute of Health, Bethesda, Maryland, United States)^17^. To measure DH/DA ratio, fundus photographs were imported to ImageJ to measure the areas. By using the polygon sections tool, margins of DH and ONH were marked, and the areas presented as pixels were recorded (Fig. 2). All measurements were performed three times and the mean of three values was used for the analyses. Two investigators (J. C. and Y. H. H.) masked to the clinical information independently evaluated the shape, type, location, layer, and the DH/DA. Discrepancies were resolved through discussion between the two investigators. If a hemorrhage spanned more than 1 clock-hour, the sector with the greatest area was selected. If multiple hemorrhages were observed within the same eye, the areas were summed and the shape, type, layer, and location of the hemorrhage with the greatest area were recorded.

### Statistical analyses

The clinical characteristics and features of DH, including age, refractive error (presented as spherical equivalent), IOP, visual field indices, and DH/DA ratio between the PVD and glaucoma groups were compared using an independent *t*-test. Distribution of sex, presence of diabetes, hypertension, shape, type, layer, and location of DH was compared using a Chi-square test or Fisher’s exact test with pairwise Z-tests for Bonferroni adjusted post hoc* analysis*. The threshold for statistical significance was set at *p* < 0.05. Statistical analyses were performed using SPSS Version 26.0 (SPSS, Chicago, IL, USA).

## Data Availability

The data are not available for public access because of patient privacy concerns, but are available from the corresponding author on reasonable request.
